# Hyperthermic Intraperitoneal Chemotherapy–Induced Molecular Changes in Humans Validate Preclinical Data in Ovarian Cancer

**DOI:** 10.1200/PO.21.00239

**Published:** 2022-03-31

**Authors:** Thanh H. Dellinger, Ernest S. Han, Mustafa Raoof, Byrne Lee, Xiwei Wu, Hyejin Cho, Ting-Fang He, Peter Lee, Marianne Razavi, Winnie S. Liang, Daniel Schmolze, Saul J. Priceman, Stephen Lee, Wei-Chien Lin, Jeff F. Lin, Mehdi Kebria, Amy Hakim, Nora Ruel, Daphne B. Stewart, Edward W. Wang, Benjamin I. Paz, Mark T. Wakabayashi, Mihaela C. Cristea, Lorna Rodriguez-Rodriguez

**Affiliations:** ^1^ Division of Gynecologic Oncology, Department of Surgery, City of Hope National Medical Center, Duarte, CA; ^2^ Division of Surgical Oncology, Department of Surgery, City of Hope National Medical Center, Duarte, CA; ^3^ Department of Surgery, Stanford University, Stanford, CA; ^4^ Integrative Genomics Core, City of Hope National Medical Center Beckman Research Institute, Duarte, CA; ^5^ Immuno-oncology Core, City of Hope National Medical Center Beckman Research Institute, Duarte, CA; ^6^ Women's Cancer Center, City of Hope National Medical Center, Duarte, CA; ^7^ Translational Genomics Research Institute, Phoenix, AZ; ^8^ Department of Pathology, City of Hope National Medical Center, Duarte, CA; ^9^ Hematology & Hematopoietic Cell Transplantation and Immuno-Oncology, City of Hope National Medical Center Beckman Research Institute, Duarte, CA; ^10^ Biostatistics Core, City of Hope National Medical Center Beckman Research Institute, Duarte, CA; ^11^ Department of Medical Oncology and Therapeutics Research, City of Hope National Medical Center, Duarte, CA

## Abstract

Hyperthermic intraperitoneal chemotherapy (HIPEC) confers a survival benefit in epithelial ovarian cancer (EOC) and in preclinical models. However, the molecular changes induced by HIPEC have not been corroborated in humans.

**PATIENTS AND METHODS:**

A feasibility trial evaluated clinical and safety outcomes of HIPEC with cisplatin during optimal cytoreductive surgery (CRS) in patients with EOC diagnosed with stage III, IV, or recurrent EOC. Pre- and post-HIPEC biopsies were comprehensively profiled with genomic and transcriptomic sequencing to identify mutational and RNAseq signatures correlating with response; the tumor microenvironment was profiled to identify potential immune biomarkers; and transcriptional signatures of tumors and normal samples before and after HIPEC were compared to investigate HIPEC-induced acute transcriptional changes.

**RESULTS:**

Thirty-five patients had HIPEC at the time of optimal CRS; all patients had optimal CRS. The median progression-free survival (PFS) was 24.7 months for primary patients and 22.4 for recurrent patients. There were no grade 4 or 5 adverse events. Anemia was the most common grade 3 adverse event (43%). Hierarchical cluster analyses identified distinct transcriptomic signatures of good versus poor responders to HIPEC correlating with a PFS of 29.9 versus 7.3 months, respectively. Among good responders, significant HIPEC-induced molecular changes included immune pathway upregulation and DNA repair pathway downregulation. Within cancer islands, % programmed cell death protein 1 expression in CD8+ T cells significantly increased after HIPEC. An exceptional responder (PFS 58 months) demonstrated the highest programmed cell death protein 1 increase. Heat shock proteins comprised the top differentially upregulated genes in HIPEC-treated tumors.

**CONCLUSION:**

Distinct transcriptomic signatures identify responders to HIPEC, and preclinical model findings are confirmed for the first time in a human cohort.

## INTRODUCTION

Epithelial ovarian cancer (EOC) is a peritoneal surface malignancy characterized by peritoneal dissemination of metastatic tumors at initial presentation and recurrence. A major treatment challenge of peritoneal surface malignancies is the poor efficacy of intravenous chemotherapies to the peritoneum. Chemotherapies such as cisplatin have higher intraperitoneal (IP) concentrations and longer half-lives in the peritoneal cavity compared with intravenous administration. This pharmacokinetic advantage translates to a significant survival benefit in randomized EOC trials.^1,2^ Nonetheless, IP therapy has not been widely adopted.^3^

**CONTEXT**

**Key Objective**
Ovarian cancer with peritoneal metastases is associated with a poor prognosis, and intraperitoneal therapies such as hyperthermic intraperitoneal chemotherapy (HIPEC) have demonstrated survival benefits in some patients. Currently, no predictive molecular criteria exist for selection of HIPEC treatment. Additionally, the molecular mechanisms of action of HIPEC are not fully elucidated. This study prospectively examined whole-transcriptomic signatures in tumors of patients with epithelial ovarian cancer undergoing HIPEC. Pre- and post-HIPEC tumors were evaluated for tumor microenvironment changes and RNAseq alterations.
**Knowledge Generated**
Whole-transcriptome sequencing demonstrated distinct signatures in HIPEC responders versus nonresponders. Comparison of tumors before and after HIPEC exposure revealed increased heat shock protein expression, immune-related pathways, and programmed cell death protein 1 expression.
**Relevance**
Use of a validated predictive RNAseq signature could allow treatment stratification of patients who would respond to HIPEC versus those who would not.

Support for hyperthermic intraperitoneal chemotherapy (HIPEC) has increased.^4^ HIPEC is the delivery of heated chemotherapy (42°C) into the IP cavity immediately after optimal cytoreductive surgery (CRS). Evidence for HIPEC is based on OVHIPEC-1, a randomized phase III trial, which demonstrated an 11.8-month overall survival benefit for patients with stage III EOC undergoing interval cytoreduction and HIPEC versus no HIPEC.^5-7^ However, questions remain regarding optimal patient selection, drug choice, toxicities, and HIPEC timing in EOC.^3,8-10^

Theoretical advantages of hyperthermia include an increased cytotoxic effect, strengthened by synergy with cisplatin through increased cellular uptake of cisplatin and improved crosslinking to DNA. Other heat-induced effects include increased vascular flow and enhanced cell membrane permeability leading to cytokine release. Additionally, heat-induced synthesis and secretion of heat shock proteins (HSPs) activate cytotoxic T lymphocytes, dendritic cells, and natural killer cells, triggering innate and adaptive immune responses.^11,12^ Other overlapping mechanisms of HIPEC include impaired DNA repair, protein denaturation, increased apoptosis, and inhibition of angiogenesis.^13,14^ Although these mechanisms have been demonstrated in preclinical studies, no human studies have investigated HIPEC-related immune pathway upregulation or DNA repair dysregulation in vivo. Furthermore, no molecular markers of response to HIPEC have been identified.^15,16^

Challenges of incorporating HIPEC into EOC treatment include optimizing HIPEC delivery with reduced associated side effects while improving identification of patients who may benefit from the treatment. We analyzed the clinical and safety outcomes of patients with EOC treated with HIPEC in a feasibility study, and we comprehensively profiled HIPEC tumors with genomic, transcriptomic, and immune microenvironment signatures to identify the characteristics of good and poor responders and investigate acute HIPEC-induced acute transcriptional changes in humans.

## PATIENTS AND METHODS

### Study Population

**FIG 1.**
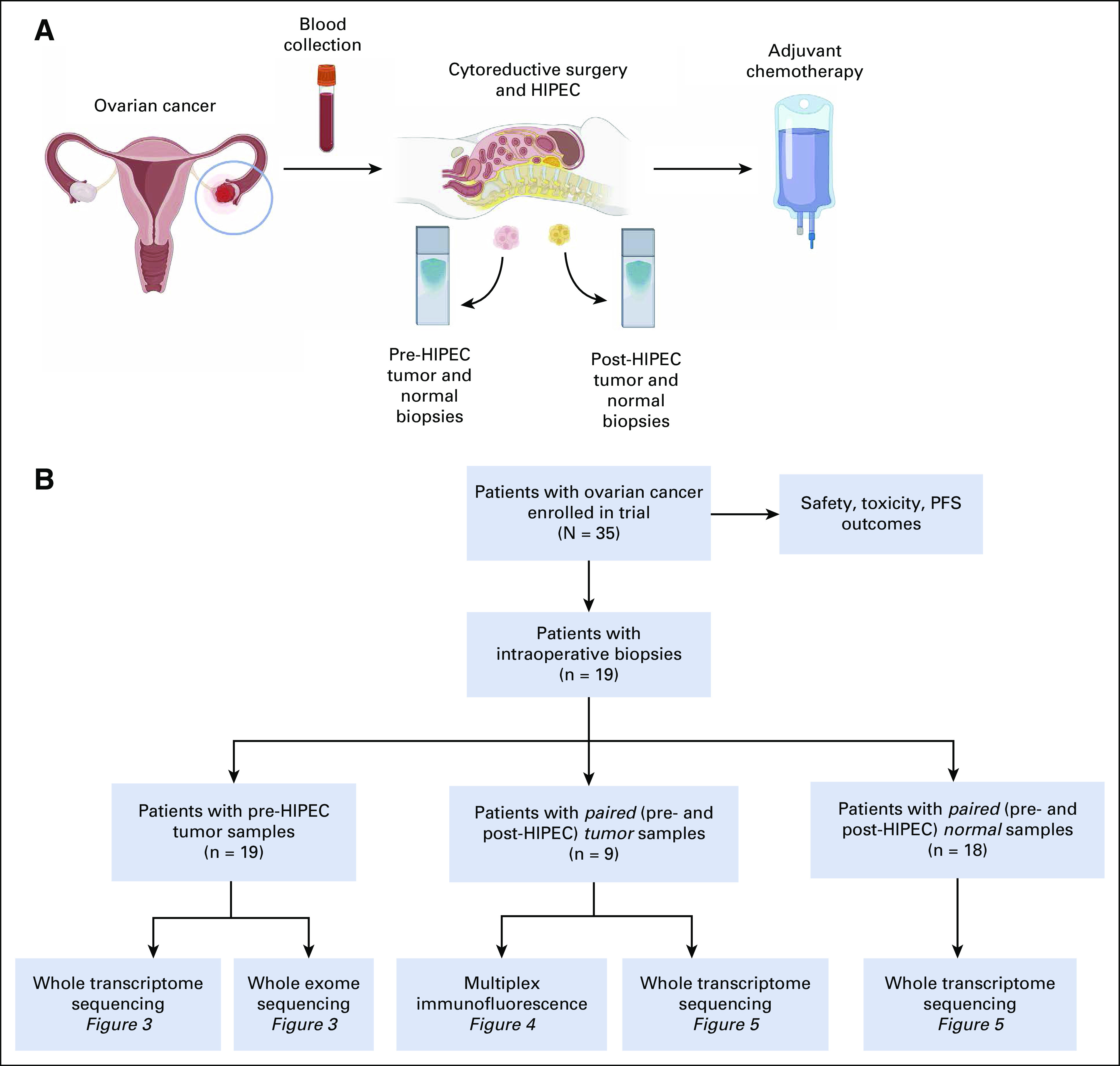
Study schema: (A) study flow and (B) flow chart of data processing and analysis. HIPEC, hyperthermic intraperitoneal chemotherapy; PFS, progression-free survival.

A single-institution clinical trial (feasibility study) evaluated the safety and feasibility of HIPEC in patients with EOC (primary end point). Progression-free survival (PFS) and correlation of clinical outcomes with molecular immune markers and genomic and transcriptomic signatures were also assessed. Patients with newly diagnosed stage III/IV EOC, primary peritoneal or fallopian tube cancer, or recurrent EOC, with an Eastern Cooperative Oncology Group performance status of 0-1, and deemed surgical candidates, who proceeded with an optimal CRS (gross residual disease < 1 cm), were eligible. An Institutional Review Board approved the trial (ClinicalTrials.gov identifier: NCT01970722). Written informed consent was obtained before patient inclusion. Patients were stratified by primary disease—HIPEC at interval CRS after neoadjuvant chemotherapy for newly diagnosed EOC; recurrent disease—HIPEC at interval or up-front CRS for recurrent EOC. Adjuvant chemotherapy was given at the discretion of the treating oncologist after HIPEC, per standard of care (Fig 1A).

Enrollment proceeded until the targeted patient number was accrued without triggering safety stopping rules (grade ≥ 4 morbidity rate ≥ 40%; mortality rate ≥ 3.4%).^17^ All recruited subjects were patients undergoing treatment at City of Hope National Comprehensive Cancer Center. Complete eligibility criteria are summarized in the Data Supplement.

Baseline demographics were obtained at enrollment. Surgery details were collected from operative notes. Peritoneal cancer index was surgeon assigned according to the Sugarbaker^18^ method. Patients were followed prospectively for evaluation of response to treatment, disease status, PFS, and mean follow-up. Platinum resistance was defined as progressive or persistent disease or progression within 6 months of completing platinum therapy.

### Hyperthermic Intraperitoneal Chemotherapy

HIPEC was delivered intraoperatively with cisplatin, 75 mg/m^2^ over 60 minutes, at 41°C-43°C immediately after optimal CRS using closed or laparoscopic methods (surgeon preference; Data Supplement). Patients were supported with filgrastim postoperatively. In January 2019, after interim analysis demonstrating acute and chronic kidney injuries, sodium thiosulfate (nephro protectant) was added as a preoperative bolus and postoperative maintenance dose, on the basis of OVHIPEC-1 dosing.^5^

### Translational Samples

Tumor and normal samples were obtained at the start of CRS (pre-HIPEC) and immediately after HIPEC (post-HIPEC). Sample collection, processing methods, and pathology assessment are presented in the Data Supplement.

### Isolation of DNA and RNA, Library Preparation, and Next-Generation Sequencing

Genomic DNA was isolated from tumor and buffy coat (QIAamp DNA mini Kit; Qiagen, Germantown, MD). RNA from formalin-fixed paraffin-embedded (FFPE; miRNeasy RNA FFPE Kit; Qiagen) and snap-frozen tissues (miRNeasy RNA mini Kit; Qiagen) was extracted following manufacturer's instructions. Concentration and purity were measured using NanoDrop One Spectrophotometer (Thermo Fisher Scientific, Waltham, MA) and Qubit 3.0 Fluorometer (Thermo Fisher Scientific).

Whole-exome library construction used 200 ng of genomic DNA per sample. Whole-transcriptome library construction used 500 ng of total RNA per sample. Methods are detailed in the Data Supplement.

### Somatic Mutations

The oncoplot function in maftools was used to draw the oncoplot of the top 20 mutated genes with histology and response annotation. Methods are detailed in the Data Supplement.

### Differential Gene Expression Analysis

Differential expression analysis comparing pre- and post-HIPEC samples identified significantly changed genes using the edgeR package v3.26.7.^19^ Pathway analysis was conducted using gene set enrichment analysis (GSEA), and GSEA analyzed upregulated or downregulated gene sets in specific groups of tumor and normal samples.^20^ A heatmap of differentially expressed genes was generated using Cluster 3.0^21^ and Java Treeview.^22^ Methods are detailed in the Data Supplement.

### Multiplex Immunofluorescence

FFPE tumor tissues were sectioned into 3-micron thickness and baked onto glass microscope slides. Multiplex immunofluorescence was performed (Opal TSA system; Akoya Biosciences, Marlborough, MA) with anti-pan cytokeratin (pan CK; clone AE1/AE3; Agilent, Santa Clara, CA), anti-CD8 (clone SP16; Biocare Medical, Pacheco, CA), anti-FOXP3 (236A/E7; Abcam, Cambridge, UK), anti-programmed cell death protein 1 (PD-1; NAT105; Cell Marque, Rocklin, CA), and anti-CD3 (polyclone; Agilent) antibodies. Methods are detailed in the Data Supplement.

### Statistical Analysis

PFS was defined as time in months from CRS and HIPEC to progression by cancer antigen-125 (Gynecologic Cancer Intergroup Criteria), imaging (computed tomography or positron emission tomography computed tomography, per RECIST), or clinical symptoms or deterioration. Kaplan-Meier and log-rank tests compared PFS of patients with low versus high %PD-1 expression changes (delta %PD-1) after HIPEC, with dichotomization on the basis of median delta %PD-1.

## RESULTS

### Clinical Characteristics

Clinical and biomarker results for 35 patients with EOC are reported (Table 1). Primary patients represented 54% of patients with pretreatment clinical stages of III (53%) and IV (47%). Recurrent patients represented 45.7%: 75% platinum-sensitive and 25% platinum-resistant. The most common histological subtype was high-grade serous (HGS) histology (69%). All primary patients with EOC underwent neoadjuvant chemotherapy (3-4 cycles) before CRS and HIPEC. Most recurrent patients (81%) did not receive neoadjuvant chemotherapy.

**TABLE 1.**
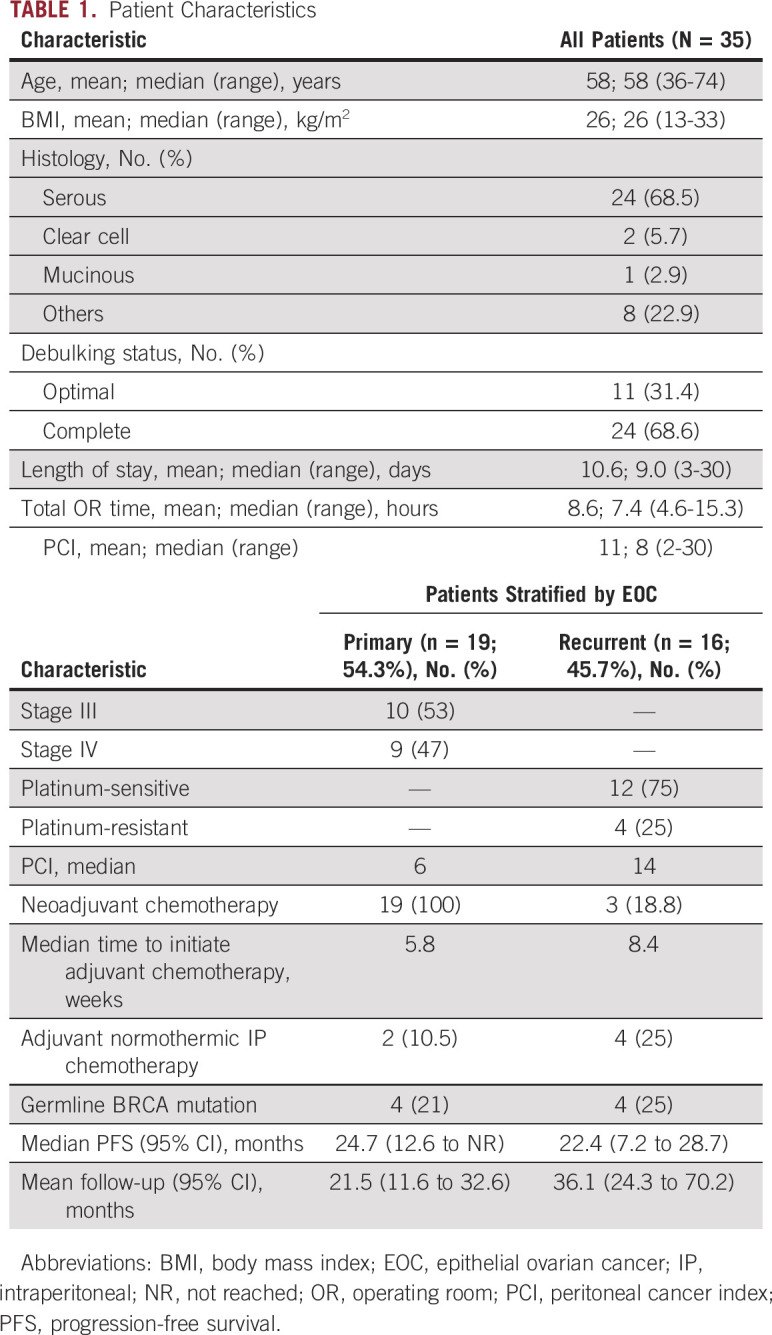
Patient Characteristics

### Clinical Outcomes

**FIG 2.**
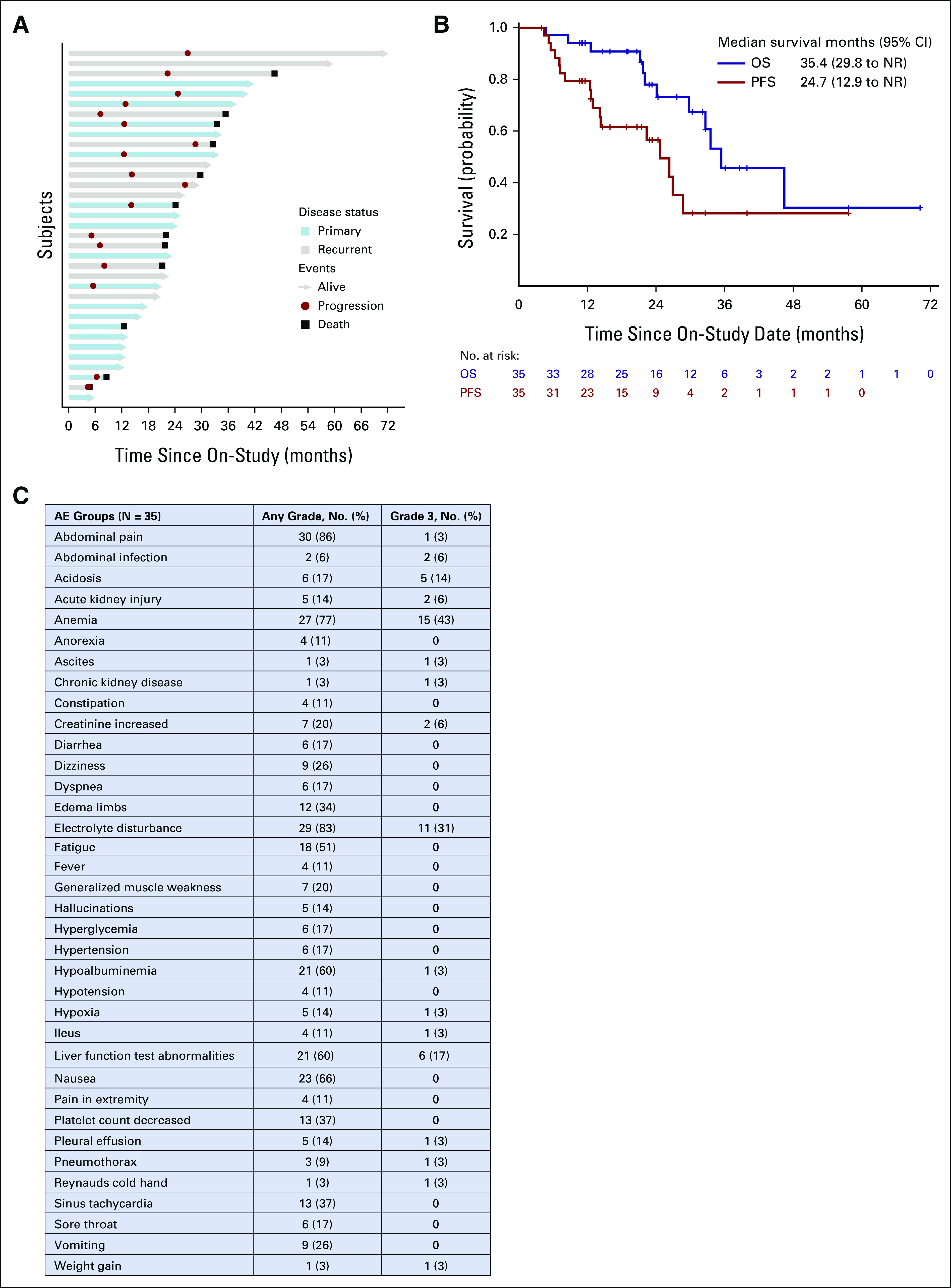
Patient outcomes (survival and safety): (A) Swimmer plot showing disease status and outcomes for all patients with a follow-up of 72 months. (B) Kaplan-Meier survival curve depicting PFS and OS for all patients. (C) AEs—treatment-related toxicity. Bars represent on-study and follow-up period. AE, adverse event; NR, not reached; OS, overall survival; PFS, progression-free survival.

All patients underwent HIPEC at the time of optimal CRS: 33 closed method and two laparoscopic. All patients underwent optimal CRS; 69% underwent complete (R0) resection (no gross residual disease). The median peritoneal cancer index was higher in recurrent vs. primary patients (14 *v* 6). The median time to initiation of postoperative chemotherapy was 7.1 weeks. Twenty-three patients (66%) had adjuvant intravenous chemotherapy; four patients (11%) had targeted or hormonal therapy; two (5.7%) recurrent patients did not undergo adjuvant chemotherapy (persistent renal failure, 1; patient choice, 1). Six (17%) patients underwent adjuvant IP chemotherapy. The median PFS for primary and recurrent patients was 24.7 (12.6 to not reached) and 22.4 (7.2 to 28.7) months, respectively. Figure 2A shows PFS for primary and recurrent patients at the 72-month follow-up. Figure 2B shows PFS and overall survival for all patients.

### Safety

There were no grade 4 or 5 adverse events (AEs; Fig 2C). Anemia was the most common grade 3 AE (43%), followed by electrolyte disturbances (31%) and liver toxicity (17%).The most common AE of any grade was abdominal pain (86%). Grade 3 acute kidney injury was observed in two patients (6%), and five patients (14%) had any grade AEs. Only one patient experienced grade 3 chronic kidney disease. After sodium thiosulfate introduction (January 2019), there were no grade 3 acute or chronic kidney injuries among 19 treated patients.

### Translational Studies

Fifty-four samples were analyzed (Fig 1B); 80% of tumor samples had at least 50% tumor cells.

### Outcome-Related Gene and Mutational Signatures

**FIG 3.**
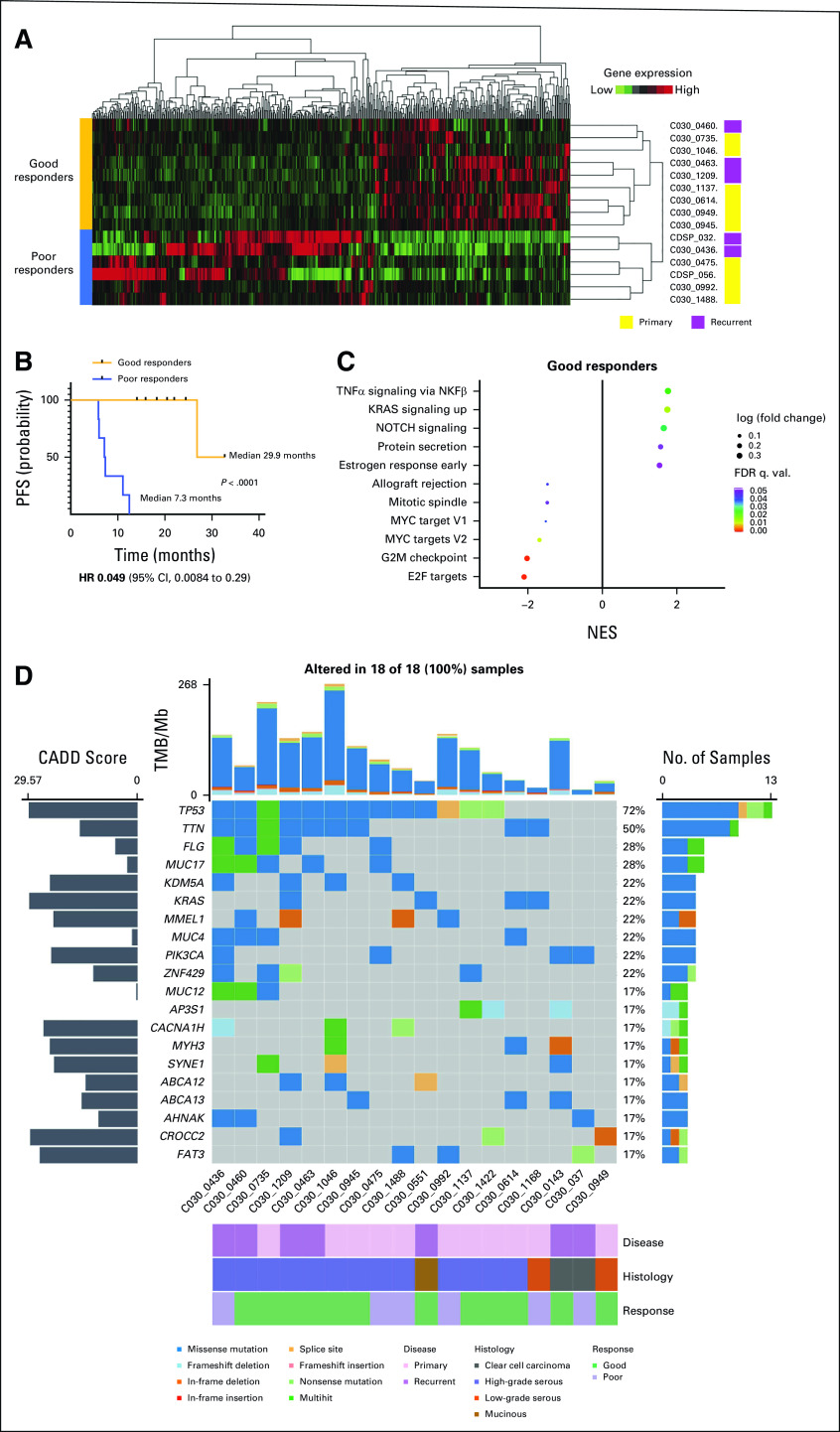
Outcome-related gene and mutational signatures of HIPEC responders. (A) Hierarchical clustered analysis of significantly changed genes in tumor samples of 15 patients. Responders were categorized into good responders (PFS > 12 months) and poor responders (PFS < 12 months). (B) Kaplan-Meier survival curve depicting PFS of good versus poor responders. (C) Kegg pathway gene set enrichment analysis from tumors of HIPEC responders demonstrate the top significantly upregulated and downregulated pathways (FDR < 0.05). Several immune-related pathways are upregulated in good responders, most prominently TNFα signaling via NFκB, while metabolic pathways are downregulated. (D) Oncoplot of the top 20 mutated genes. The upper bar plot indicates the number of intergenic somatic variants per patient while the right bar plot shows the number of variants per gene. The CADD score is shown on the left. The mutation types, histology types, and response to HIPEC are noted below the oncoplot. CADD, combined annotation dependent depletion; FDR, false discovery rate; HIPEC, hyperthermic intraperitoneal chemotherapy; HR, hazard ratio; NES, normalized enrichment score; NFκB, nuclear factor kappa B; PFS, progression-free survival; q. val., q value; TMB, tumor mutational burden; TNFα, tumor necrosis factor α.

Whole-transcriptome sequencing (WTS) identified correlations between survival and gene expression in tumor samples from 15 patients with available WTS and survival data > 12 months. Responders were categorized into good (PFS > 12 months) and poor (PFS < 12 months). Supervised clustering analyses identified distinct WTS signatures for different responders (Fig 3A). Good responders demonstrated superior PFS compared with poor responders; 29.9 versus 7.3 months (Fig 3B). Among good responders, tumor necrosis factor (TNF)α signaling via nuclear factor kappa B (NFκB), KRAS signaling, and Notch signaling were the most upregulated Kegg GSEA pathways; downregulated pathways included E2F targets, G2M checkpoint pathways, and Myc targets (Fig 3C). Predictive molecular signatures in patients with recurrent EOC are summarized in the Data Supplement.

### Genomic Alterations Associated With HIPEC Response in Pretreatment Tumor Biopsies

Mutational signatures present in pre-HIPEC tumor samples (n = 19) were examined by deconvoluting the frequency of the 96 different possible trinucleotide substitutions against known signatures of mutation patterns. DNA repair signatures were equally present in all responders, demonstrating no significant correlation with clinical outcomes (Data Supplement).The oncoplot of the top 20 mutated genes with disease, histology, and response is shown in Figure 3D. Of 19 patient tumors subjected to whole-exome sequencing, one patient sample did not exhibit nonsynonymous mutations and was excluded in the oncoplot analysis. For the 18 remaining tumors, TP53 was the most commonly altered gene. KRAS and PIK3CA were altered in 22% of samples. Tumors from four patients exhibited KRAS mutations, of which three were associated with a good response. Copy number alterations are summarized in the Data Supplement.

### Tumor Microenvironment Changes After HIPEC

**FIG 4.**
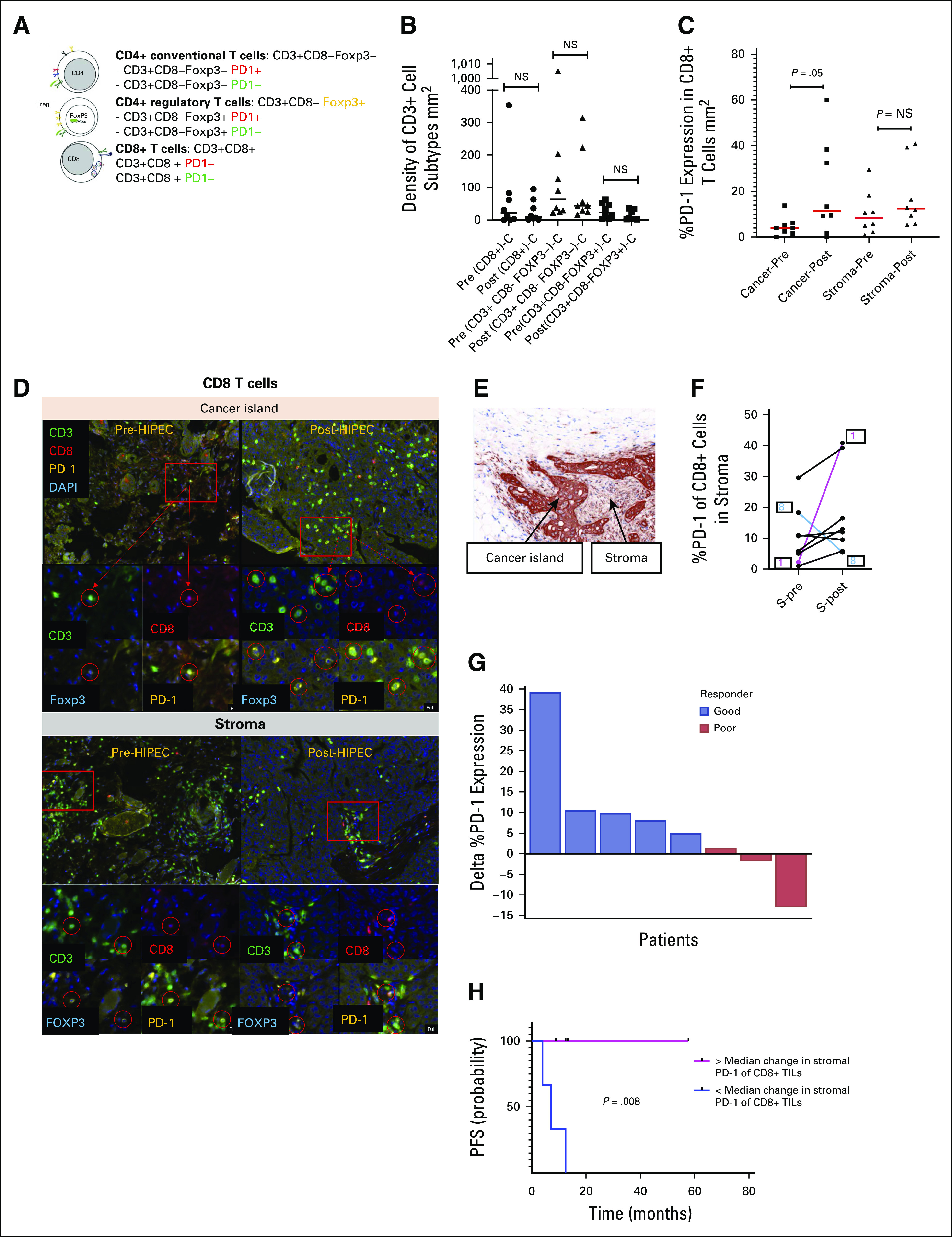
Tumor microenvironment changes induced by HIPEC. Multiplex immunofluorescence estimation of tumor-infiltrating immune subsets and PD-1 expression in matched pre- and post HIPEC tumors. (A) Pre- and post-HIPEC tumors were stained with TIL markers (CD3, CD8, FOXP3), PD-1, for the following phenotypes: CD4+ conventional T cells (CD3+ CD8– FOXP3– cells), CD4+ regulatory T cells (CD3+ CD8– FOXP3+), and CD8+ T cells (CD3+ CD8+). (B) Cell density of CD8+ T cells, CD4+ conventional T cells, and CD4+ regulatory T cells do not change with HIPEC treatment. (C) Evaluation of PD-1 expression in CD8+ T cells. PD-1 expression rises in CD8+ T cells within cancer islands (cancer), but not in stroma. (D) Representative immunofluorescence demonstrating increased PD-1 staining of CD8+ T cells within cancer islands after HIPEC while stromal PD-1 expression remained stable after HIPEC. (E) CK was used as a marker for cancer islands to delineate it from stroma. (F) PD-1 expression changes in individual patients before and after HIPEC in CD8+ T cells (stroma). The patient with the highest rise in %PD-1 was patient 1, an exceptional responder with PFS of 5 years and demonstrated the largest PD-1 increase. The only patient with decreased PD-1 expression was patient 8, who was the only patient with clear cell histology and had a poor survival (PFS < 12 months). (G) PD-1 expression changes (delta %PD-1) after HIPEC and correlation with response. %PD-1 expression changes are denoted per patient (patients 1-8). Responders were categorized into good (PFS > 24 months) and poor (PFS < 12 months). Poor responders have negative PD-1 expression changes while good responders have positive PD-1 expression changes, with an exception responder (patient 1) demonstrating the largest PD-1 rise. (H) Kaplan-Meier curves of PFS in patients with high (pink) versus low (blue) delta %PD-1 expression changes in CD8+ TILs. %PD-1 expression was dichotomized on the basis of a median delta %PD-1 threshold. CK, cytokeratin; DAPI, 4',6-diamidino-2-phenylindole; HIPEC, hyperthermic intraperitoneal chemotherapy; NS, not significant; PD-1, programmed cell death protein 1; PFS, progression-free survival; TIL, tumor-infiltrating lymphocyte.

Multiplex immunofluorescence evaluated T-cell density and %PD-1 expression after HIPEC within cancer islands and stroma of CD8+ T cells and CD4+ conventional T cells (Fig 4A). Metastatic tumors with matched pre- and post-HIPEC samples (nine patients) were stained with CD3, CD8, FOXP3, PD-1, and pan CK; pan CK represents cancer island staining (Fig 4E). CD8+, CD4+, and CD4+ regulatory T-cell density did not change after HIPEC (Fig 4B). Within cancer islands, %PD-1 expression in CD8+ T cells significantly increased after HIPEC (Fig 4C). Representative immunofluorescence demonstrated increased PD-1 staining of CD8+ T cells within cancer islands while stromal PD-1 expression remained stable (Fig 4D). Individual patient %PD-1 expression changes in CD8+ T cells showed that the patient with the highest rise in %PD-1 was an exceptional responder (patient 1, PFS of 58 months; Fig 4F). The only patient with substantially decreased PD-1 expression had poor survival (PFS < 12 months) and clear cell histology (patient 8).

The magnitude of %PD-1 expression change in CD8+ T cells within stroma correlated with response to HIPEC (Fig 4G). For this analysis, responders were categorized into good (PFS > 24 months) and poor (PFS < 12 months). Good responders exhibited high PD-1 expression increases in CD8+ T cells within stroma while poor responders exhibited minimal or negative PD-1 expression changes (Fig 4G). Patients 1 and 8 had recurrent EOC; HIPEC-induced PD-1 expression changes were associated with differential PFS curves.

%PD-1 expression changes were categorized into high versus low delta %PD-1 expression groups. Patients with high %PD-1 expression changes had superior PFS compared with those with low %PD-1 expression changes (Fig 4H).

Similar to CD8+ tumor-infiltrating lymphocytes, CD4+ conventional T cells exhibited increased %PD-1 expression after HIPEC, with similar response associations for exceptional and poor responders (Data Supplement).

### HIPEC-Induced Differentially Expressed Gene Analysis and Pathway Changes

**FIG 5.**
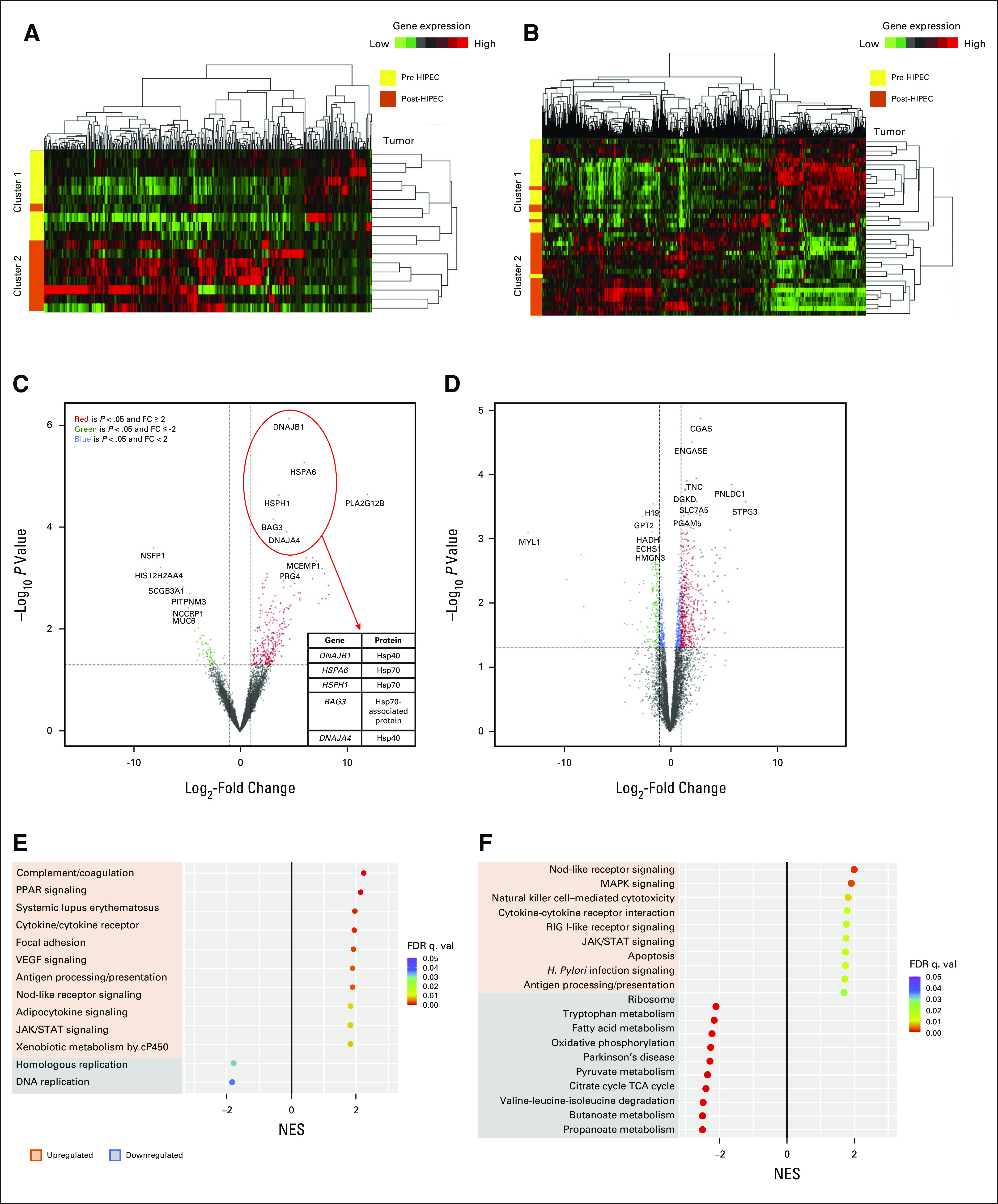
Gene and pathway changes of pre- and post-HIPEC in metastatic tumor and normal patient samples. (A and B) Hierarchical clustered analysis of (A) differentially expressed genes in metastatic tumors and (B) normal samples. Cluster 1 was enriched for pre-HIPEC; cluster 2 was enriched with post-HIPEC. (C) Volcano plot displaying gene expression post-HIPEC by log_2_-fold change (*x*-axis) and minus log_10_ *P* value (*y*-axis) in tumor samples. Significantly upregulated genes of ≥ 2-fold expression are in red while statistically significantly downregulated genes of ≥ 2-fold expression are in green. The top five differentially expressed genes with fold change (post- *v* pre-HIPEC) and *P* value shown for tumor samples are shown. (D) Volcano plot displaying gene expression post-HIPEC by log_2_-fold change (*x*-axis) and minus log_10_ *P* value (*y*-axis) in normal samples. (E and F) Kegg pathway gene set enrichment analysis of (E) HIPEC metastatic tumors and (F) normal samples demonstrating the top significantly upregulated and downregulated pathways (FDR < 0.05). Several immune-related pathways are upregulated, and DNA replication pathways are downregulated in tumor samples. Normal HIPEC samples demonstrate significant downregulation of metabolic pathways. FC, fold change; FDR, false discovery rate; HIPEC, hyperthermic intraperitoneal chemotherapy; NES, normalized enrichment score; q. val., q value; TCA, tricarboxylic acid.

Histologic changes after HIPEC that differed between tumors and normal tissues are shown in the Data Supplement. Genes of pre- and post-HIPEC metastatic tumors, analyzed by using hierarchical clustered analysis (Fig 5), demonstrated two clusters: 1—enriched in pre-HIPEC tumors; 2—only in post-HIPEC tumors (Fig 5A). Similarly, in normal tissues, cluster 1 was enriched in pre-HIPEC normal samples and cluster 2 in post-HIPEC normal samples (Fig 5B). Volcano plots show significantly upregulated and downregulated HIPEC-induced gene changes (Figs 5C and 5D). Greater HIPEC-induced gene changes were seen in tumor versus normal samples (wider angle of volcano plot in tumors).The top differentially expressed genes in pre- and post-HIPEC tumors are shown in Figure 5C. Five HSPs were among the top six most significantly upregulated genes in tumors after HIPEC. Among normal samples, top differentially expressed genes correlated primarily with metabolism (Fig 5D).

Metastatic tumors demonstrated significant upregulation in immune-related pathways after HIPEC (Fig 5E). DNA repair and homologous replication pathways were downregulated in tumors. In normal tissues, metabolic pathways were downregulated and immune-related pathways were upregulated to a lesser extent compared with tumors (Fig 5F).

## DISCUSSION

Despite enthusiasm for HIPEC in EOC, questions remain regarding optimal patient selection and, specifically, identification of patients who may not benefit from therapy. Additionally, translational research to elucidate the immune and tumor microenvironment (TME) effects of HIPEC has been limited. We present the first prospective study to comprehensively profile HIPEC-treated EOC patients' tumors with genomic, transcriptomic, and TME analyses to identify signatures correlating with survival and elucidate early molecular changes induced by HIPEC.

To the best of our knowledge, this is the first report identifying immune biomarkers and gene signatures associated with response to HIPEC in EOC. Our study used WTS to identify unique gene signatures for good vs. poor responders to HIPEC. Good responders showed the greatest upregulation in TNFα signaling via the NFκB pathway, a pathway that appears to play a role in platinum resistance and sensitivity.^23^ Additionally, NFκB plays a major role in B-cell and T-cell activation. Upregulation of TNFα signaling via the NFκB pathway in good responders to HIPEC may reflect the increased systemic inflammatory response seen after cisplatin-based HIPEC treatments.^24^ The second greatest upregulated gene pathway among good responders was the KRAS signaling pathway, a pathway predominantly seen in type II (non-HGS) ovarian cancer tumors. Among genomic alterations in the patient cohort, only four patients exhibited KRAS mutations.

We demonstrated HIPEC-induced T-cell activation through expression of PD-1. Although the PD-1 pathway is widely known for its role in T-cell exhaustion and tumor immunosuppression, PD-1 is not an exhaustion-specific marker as it is also expressed on all conventional CD4+ T cells and CD8+ T cells during acute T-cell activation.^25^ Given the acute setting of hyperthermic cisplatin exposure and short duration of a few hours between the collection of pre- and post-HIPEC tumors, we interpret the immediate PD-1 expression after HIPEC as a marker of T-cell activation. These findings imply a role for HIPEC in modulating the TME. Moreover, we demonstrate that the magnitude of PD-1 surge immediately after HIPEC correlates with improved PFS, suggesting an opportunity for future biomarker study. Given these findings, further study on the role and timing of PD-1 inhibition in HIPEC treatment is -the best of our knowledge, our study reveals for the first time the in vivo effects of HIPEC on the release of HSPs and the resultant inflammatory and immunogenic processes (Data Supplement). We showed that Hsp40, Hsp70, and Hsp70-associated proteins were the most upregulated genes in post-HIPEC tumors. In vitro studies have demonstrated the effect of hyperthermia on Hsp70, which is released from tumor cells during necrosis. In extracellular form, Hsp70 interacts with receptors on inflammatory cells, causing a secondary release of inflammatory cytokines and nitric oxide from monocytes and macrophages, thus exerting a profoundly proinflammatory effect.^26^ We observed upregulation of several immune-associated and inflammatory pathways, including the antigen processing/presentation pathway. This concurs with preclinical studies indicating that HSPs activate dendritic cells transforming them into mature antigen-presenting cells.^27^ Similarly, our report of upregulated cytokine/cytokine receptor pathways reflects preclinical studies demonstrating release of cytokines as a result of hyperthermia-augmented cell membrane permeability, resulting in enhanced drug penetration into tumor cells.^28^ Finally, our analysis revealed HIPEC-induced downregulation of DNA replication pathways, which is consistent with preclinical studies of hyperthermia and cisplatin interfering with DNA repair response cascades, resulting in nuclear protein denaturation, hampered DNA repair during the S phase, resultant DNA relaxation and clumping, and eventual mitotic catastrophe.^29,30^

We demonstrated the safety and feasibility of HIPEC and cisplatin in primary and recurrent patients with EOC. No grade 4 or 5 AEs were reported; the most common grade 3 AEs (anemia, liver toxicity, and electrolyte disturbances) are those expected from CRS. In contrast, the OVHIPEC-1 study reported abdominal pain, ileus, and infection as the most common grade 3 or 4 AEs, which are primarily surgical sequelae. Renal toxicities were significant only in patients without sodium thiosulfate use. Similarly, in the OVHIPEC-1 study, no significant renal toxicities were reported with the use of sodium thiosulfate.

The PFS after HIPEC in primary patients with stage III and IV EOC was 24.7 months, longer than reported in OVHIPEC-1 (PFS of 14.2 months for patients with stage III EOC).^5^ Both our study and OVHIPEC-1 used cisplatin, although our trial dose was lower (75 mg/m^2^ *v* 100 mg/m^2^) and for a shorter duration (60 *v* 90 minutes). Our study also demonstrated a median PFS of 22.4 months in recurrent patients with EOC. This exceeds the PFS from a recent US phase II trial in platinum-sensitive recurrent patients with EOC, where PFS in the carboplatin HIPEC arm was 12.3 months.^31^ Differences between these studies include the inclusion of both platinum-sensitive and platinum-resistant patients and the use of cisplatin, rather than carboplatin.

Study strengths include the extensive biomarker analyses including whole-exome and whole-transcriptome sequencing, as well as characterization of TME changes after HIPEC. Study weaknesses include a small sample size with a heterogeneous population comprising both primary and recurrent patients with EOC with both HGS and non-HGS histologies. Additionally, cisplatin dose and HIPEC duration were lower in our study than in OVHIPEC-1; nonetheless, PFS for both primary and recurrent patients was longer in our study than compared with recent prospective randomized studies.

In conclusion, our findings demonstrate that HIPEC is safe for patients with EOC and, it plays a role in modulating the TME, inhibiting DNA repair, and upregulating HSPs and subsequent immune-associated pathways. Although these mechanisms have been demonstrated in preclinical studies, to our knowledge, this is the first study to demonstrate these processes in human samples. Moreover, our study suggests the existence of potential immune biomarkers and transcriptomic signatures, which may predict survival. Larger studies are needed to further elucidate predictive biomarkers in HIPEC.

## Data Availability

A data sharing statement provided by the authors is available with this article at DOI https://doi.org/10.1200/PO.21.00239.
